# Editorial for Special Issue “Image Analysis and Machine Learning in Cancers”

**DOI:** 10.3390/cancers17050778

**Published:** 2025-02-25

**Authors:** Helder C. R. Oliveira, Arianna Mencattini

**Affiliations:** 1 School for Advanced Digital Technology, Southern Alberta Institute of Technology (SAIT), Calgary, AB T2M 0L4, Canada; 2 Department of Electronic Engineering, University of Rome Tor Vergata, 00133 Rome, Italy; mencattini@ing.uniroma2.it

Cancer detection has been a great challenge in many fields of science. According to the World Health Organization (WHO), the cancers with the highest mortality rate are lung, colorectal, liver, breast and stomach cancers [1]. It is well known that to mitigate these deaths, earlier detection is the key to increasing treatment efficacy. Manual analyses of the patient’s images are currently employed. This is subject to human error, and is also time-consuming and very expensive, costing patients’ lives. With the advent of powerful computers, doctors and patients have benefited from the techniques developed over the years that automate the assessment of suspicious regions, providing an accurate and unbiased “second” opinion.

This Special Issue has shown state-of-the-art methods for detecting various types of cancers. Using clinical data and different image modalities, such as SPECT/CT, histopathologic images, and CT, among others, the methods proposed here have shown great potential in helping clinicians and patients.

AlGhamdi et al. addressed the automatic detection of lung and colon cancer from histopathological images [2]. As in many other fields of diagnosis by imaging, manual evaluation consists of a human specialist carefully analyzing microscopic images of samples of suspicious tissue to detect cancerous cells. This task is very tedious, and prone to error. In this paper the authors introduce a new deep-learning model that has been fine-tuned using features from a large dataset of histopathological images extracted with ShuffleNet. The parameter optimization is performed using Al-Biruni Earth Radius Optimization, and the experimental results showed enhanced accuracy and reliability compared to conventional methods.

The study by Klontzas et al. focused on detecting renal oncocytic tumors [3]. The method proposed merges ^99*m*^Tc Sestamibi SPECT/CT imaging with radiomics in XGBoost classifiers to differentiate benign and malignant renal tumors. The results showed a 95% accuracy and 98.3% AUC, and it outperformed models based solely on imaging or radiomics, showcasing the crucial role of SPECT/CT in diagnostic accuracy.

Alamgeer et al. proposed a new deep feature fusion model using Dung Beetle optimization for lung cancer detection and classification [4]. The model uses features extracted with three popular deep learning models (namely ResNet, DenseNet, and Inception-ResNet-v2) whose hyperparameters are optimized via Dung Beetle Optimization. An LSTM network enhances classification, showing superior performance in lung cancer detection across benchmark datasets.

The detection and assessment of skin lesions is essential for the diagnosis of skin cancer. In this sense, the study by Dzieniszewska et al. focused on an improved lesion detection mechanism [5]. Given the lack of data for this segmentation problem, the paper proposes a self-training approach using a Noisy Student framework and DeepLabV3. By leveraging pseudo-labeling and limited labeled data, the method achieves mIoU scores of 88.0% and 87.54% on the ISIC 2018 and PH2 datasets, respectively.

Grading cells suspected of being cancerous is another task that clinicians often perform. Alhussaini et al. proposed a machine learning model to automatically grade renal cell carcinomas according to the WHO/ISUP [6]. The method has employed 3D segmentation followed by feature extraction. In total, 11 machine learning models were evaluated, reaching a 95% area under the ROC curve.

Hill et al. focused on simplifying the application of optical coherence tomography (OCT) to examine subsurface morphology in the oral cavity, to enhance its adoption as a biopsy guidance tool [7]. A total of four deep-learning models were used to compose a processing pipeline. The models achieved high performance, with an AUC of 100% for image detection, 94% for artifacts detection, and Dice similarity scores of 0.98 and 0.83 for surface and boundary segmentation, respectively.

Another study focusing on lung cancer was proposed by Vinhas et al. [8]. Since the cancer detection methods nowadays involve painful and/or invasive techniques, the Volatile Organic Compound (VOC) is a great alternative to overcome these challenges. This paper has shown a machine learning approach to automate the analysis of VOCs towards lung cancer detection. The results reported showed that AI-driven techniques have great potential for early cancer detection in a clinical setting.

In the same vein of non-invasive cancer screening, the paper by Švecová et al. proposed the use of the Urinary Fluorescent Metabolome Profile to detect endometrial cancer [9]. Using clinical data, classical machine learning models were trained, achieving an accuracy of 79% and an area under the ROC curve of 90%.

A comparative analysis of deep learning models for the detection of lung cancer is presented in [10]. Pre-trained CNNs, such as MobileNetV2, ResNet152V2, InceptionResNetV2, Xception, VGG-19, and InceptionV3, were evaluated using CT images to segment the area where the lesion is. Among the results, the researchers have found that the InceptionResNetV2 model achieved the highest accuracy of 98.5%, while UNet was best for segmentation results, with a Jaccard index of 95.3%.

Another method for lung and colon cancer detection was introduced by Ochoa-Ornelas et al. [11]. In this paper, the researchers used histopathologic images to develop a hybrid model composed of deep learning and machine learning models for the classification of Colon Adenocarcinoma, Colon Benign Tissue, Lung Adenocarcinoma, Lung Benign Tissue, and Lung Squamous Cell Carcinoma. After a sequence of imaging processing and feature extraction steps, the model proposed achieved a classification accuracy of 94.8%.

In conclusion, the papers published in this Special Issue have successfully pushed the boundaries of automation techniques towards more accurate and precise cancer detection. These methods have shown the potential of AI models that can be implemented in clinical practice, alleviating the burden on the doctors and saving patients’ lives.
